# Supplementary material for: Johnson-noise-limited cancellation-free microwave impedance microscopy with monolithic silicon cantilever probes
Source: Nat Commun. 2024 Jun 13;15:5043. doi: 10.1038/s41467-024-49405-8 (PMC11176329; doi:10.1038/s41467-024-49405-8)
Supplement: Supplementary file 1 — Supplementary Information [file 41467_2024_49405_MOESM1_ESM.pdf]

Supplementary Information for
Johnson-noise-limited cancellation-free microwave impedance microscopy with monolithic silicon cantilever probes

Jun-Yi Shan,[1,2] Nathaniel Morrison,[1,2] Su-Di Chen,[1,2,3] Feng Wang,[1,2,3] and Eric Y. Ma[1,2,4]

[1]*Department of Physics, University of California, Berkeley, Berkeley, CA 94720, USA*

[2]*Lawrence Berkeley National Laboratory, Berkeley, CA 94720, USA*

[3]*Kavli Energy NanoScience Institute, University of California, Berkeley, Berkeley, CA 94720 USA*

[4]*Department of Electrical Engineering and Computer Sciences, University of California, Berkeley, Berkeley, CA 94720, USA*

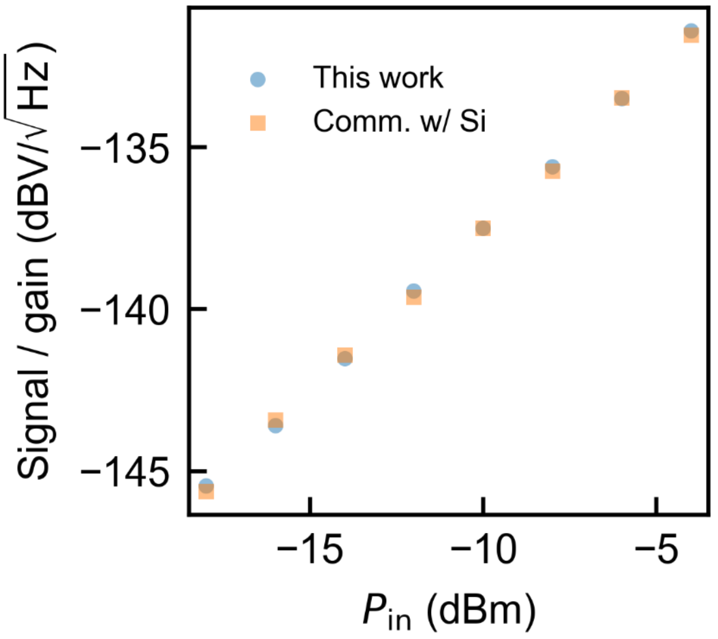

Supplementary Fig. 1. The scaling of dynamic MIM signal with P_{in}. Normalized peak amplitude at f_0 which represents the dynamic MIM signal, measured as a function of P_{in} for our cancellation-free electronics and a commercial system with multi-stage cancellation. The linear dependence shows the absence of saturation in the signal chain.

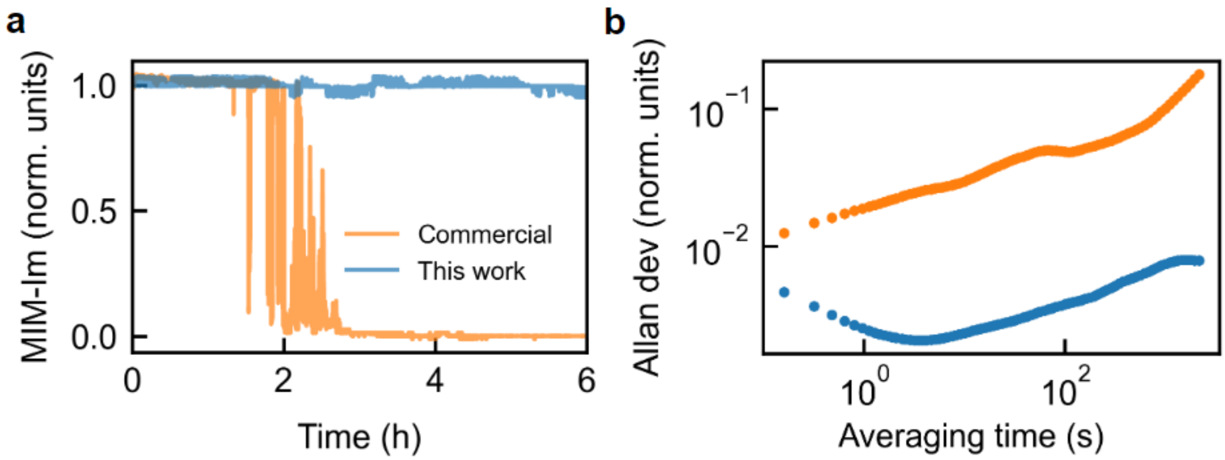

Supplementary Fig. 2. Drift of the commercial system. a, The time series of the normalized dynamic MIM signal. For the commercial and cancellation-free systems, measurements were taken at the same Al spot with intervals of 160 ms. **b**, The calculated Allan deviation for both time series. The Allan deviation of the commercial system at long time scales is more than an order of magnitude larger than that of our cancellation-free system.

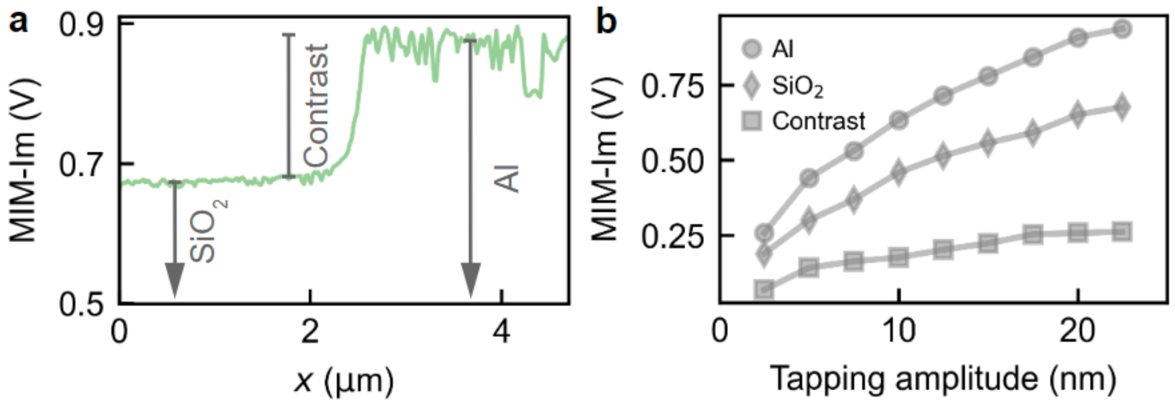

Supplementary Fig. 3. Raw and contrast signal and their dependence on cantilever vibration amplitude. a, The one-dimensional cut of an MIM-Im image taken on an Al dot sample, showing the raw Al and SiO_2 signals as well as their contrast. **b**, The MIM-Im signal as a function of peak-to-peak cantilever vibration amplitude.
